# Essential Fatty Acids as Biomedicines in Cardiac Health

**DOI:** 10.3390/biomedicines9101466

**Published:** 2021-10-14

**Authors:** Igori Balta, Lavinia Stef, Ioan Pet, Tiberiu Iancu, Ducu Stef, Nicolae Corcionivoschi

**Affiliations:** 1 Bacteriology Branch, Veterinary Sciences Division, Agri-Food and Biosciences Institute, Belfast BT4 3SD, UK; igori.balta@gmail.com; 2 Faculty of Animal Science and Biotechnologies, University of Agricultural Sciences and Veterinary Medicine, 400372 Cluj-Napoca, Romania; 3 Faculty of Bioengineering of Animal Resources, Banat University of Animal Sciences and Veterinary Medicine—King Michael I of Romania, 300645 Timisoara, Romania; Lavi_stef@animalsci-tm.ro (L.S.); ioanpet@eurofins.com (I.P.); 4 Faculty of Management and Rural Development, Banat University of Animal Sciences and Veterinary Medicine—King Michael I of Romania, 300645 Timisoara, Romania; iancu_tibi@yahoo.com; 5 Faculty of Food Processing Technologies, Banat University of Animal Sciences and Veterinary Medicine—King Michael I of Romania, 300645 Timisoara, Romania

**Keywords:** cardiovascular diseases, essentially fatty acids, fatty acid sources, cardiac health, molecular mechanisms, PFA, omega-3, EPA and DHA, symptom relief

## Abstract

The destructive impact of cardiovascular diseases on health, including heart failure, peripheral artery disease, atherosclerosis, stroke, and other cardiac pathological conditions, positions these health conditions as leading causes of increased global mortality rates, thereby impacting the human quality of life. The considerable changes in modern lifestyles, including the increase in food intake and the change in eating habits, will unavoidably lead to an unbalanced consumption of essential fatty acids, with a direct effect on cardiovascular health problems. In the last decade, essential fatty acids have become the main focus of scientific research in medical fields aiming to establish their impact for preventing cardiovascular diseases and the associated risk factors. Specifically, polyunsaturated fatty acids (PUFA), such as omega 3 fatty acids, and monounsaturated fatty acids from various sources are mentioned in the literature as having a cardio-protective role, due to various biological mechanisms that are still to be clarified. This review aims to describe the major biological mechanisms of how diets rich in essential fatty acids, or simply essential fatty acid administration, could have anti-inflammatory, vasodilatory, anti-arrhythmic, antithrombotic, antioxidant, and anti-atherogenic effects. This review describes findings originating from clinical studies in which dietary sources of FAs were tested for their role in mitigating the impact of heart disorders in human health.

## 1. Introduction

Cardiovascular diseases (CVD) are considered to be responsible for approximately 17.3 million deaths every year, accounting for 25% of the total deaths in developing countries [1]. In Western countries, over the last 150–180 years, a ten-fold increase in CVD cases has been recorded, compared to only a two-fold increase in cancer cases [2]. This increase in deaths and cases is associated with the pathophysiological patterns of CVD, which are revealed by several risk factors, such as the increased expression of pro-inflammatory cytokines, blood pressure anomalies, endothelial dysfunction, atherosclerosis, and impaired glucose metabolism, among others.

**Figure 1:**
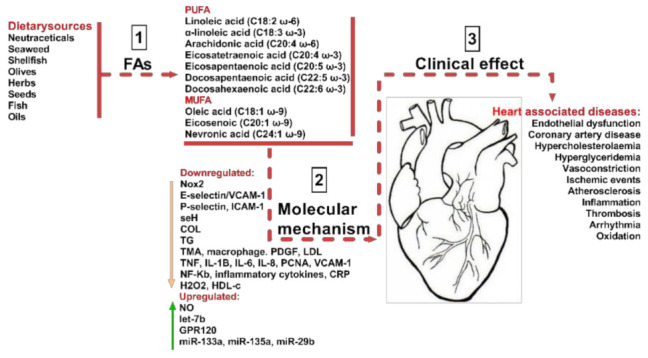
Sources of beneficial MUFA and PUFA and their impact on CVDs. As indicated in step (1) the most common dietary sources are neutraceticals, seaweed, shellfish, olives, herbs, seeds, fish, and various oils. They provide various polyunsaturated fatty acids (PUFA) including linoleic acid, arachidonic acid, eicosatetraenoic acid, eicosapentaenoic acid, docosapentaeinoic acid, and docosahexaenoic acid. Dietary sources also provide monounsaturated fatty acids (MUFA) including ω-9 oleic acid, eicosenoicacid, and nevronic acid. By various molecular mechanisms (2), they have a beneficial impact on CVD clinical effects, as described in step (3). The molecular mechanisms are Nox2-NADPH oxidase 2, E-selectin-leukocyte endothelial adhesion molecule, cell adhesion molecule, seH-lipid signaling molecule, COL-collagen factor, TG-triglycerides, TMA-thrombotic microangiopath, PDGF-platelet derived growth factor, LDL-low density lipoprotein, TNF-tumor necrosis factor, IL8, 6 and 1B-interleukins, PCNA-proliferating cell nuclear antigen, VCAM1-vascular cell adhesion protein 1, NF-Kb-nuclear factor kappa light chain enhancer of activated B cells, CRP-C reactive protein, H2O2-hydrogen peroxide, HDL-high density lipoprotein, NO-nitric oxide, let-7b-tumor suppressor miRNA, ω-3 fatty acid receptor, miR-133a, miR-135a, and miR-29b-non-coding RNAs important in cardiac muscle development.

As shown in Figure 1 a balanced diet may exert cardio-protective effects and, as such, improve human health. Previously, in the mid-19th century, our ancestors consumed diets composed mainly of wild plants, berries, fruits, vegetables, lean meat, fish, and only a few types of cereals. However, following the industrial and agricultural revolution, dietary behaviors shifted considerably, as cereals became one of the most affordable part of the diet [3]. As a result, an increased consumption of omega-6 (ω-6) fatty acids (FAs) was observed, with a substantial decrease in omega-3 (ω-3) FAs and antioxidants, caused, as was previously observed, by an increase in green leafy sources. This reduction in the levels of beneficial FA intake, such as ω-3 PUFA and MUFA, and associated with an increase in large carbohydrate consumption, has raised the progression rate of hypertension, coronary heart diseases, insulin resistance, hyperinsulinemia and obesity [3]. For example, the modification of food intake of ω-6 to ω-3 fatty acids (FAs) from 1:1 to 15–20:1 led to the induction of metabolic changes, described as being responsible for elevated levels of pro-inflammatory mediators and for the alteration of various signals, and as such contributing to the pathophysiological responses that produce CVD, diabetes Mellitus, and in some cases mental disorders (Figure 1).

Essential fatty acids (EFAs) intake varies significantly due to changes in individual food intake levels and eating behaviors, which are contributing factors to the occurrence of cardiovascular related health issues [3]. Fatty acids are essential building molecules for cellular structures, tissues, and organs and are also involved in the production of essential biologically active substances, as well in coordinating the proper function of metabolic processes [1]. Dietary fats (FAs) play a key role in the metabolic regulation of the whole human body. They are classified according to their degree of saturation and chemical structure into three distinct classes: saturated fatty acids (SFA), monounsaturated fatty acids (MUFA), and polyunsaturated fatty acids (PUFA). Each one of these classes have different physiological functions within the body, whereby MUFA and PUFA are associated with the induction of positive effects by the modulation of metabolic processes essential for the proper function of the cardiovascular system [4,5]. At the same time, SFAs are equally linked to metabolic dysfunction in the absence of a satisfactory supply [6]. Moreover, FAs generate an enormous source of adenosine triphosphate (ATP), and the β-oxidation of FAs is used by the heart and muscular tissues to obtain myocardial energy [7,8].

In vitro and in vivo experimental studies have indicated that essential fatty acids (EFAs) are, indeed, important metabolites and they have been extensively studied in the field of cardiac medicine, with remarkable outcomes aimed at improving CVDs [1]. Despite this progress, there are still unanswered questions about the molecular mechanisms of action, effective dosage, and their exact physiological ability that prevents associated cardiovascular diseases. Our approach in this review was to bring together scientific and clinical data on cardiovascular diseases and associate it with the bioavailability of FAs in different dietary sources. Our approach is summarized in Figure 1.

## 2. Biological Mechanisms of PUFA Associated with Cardio-Protection

**Figure 2:**
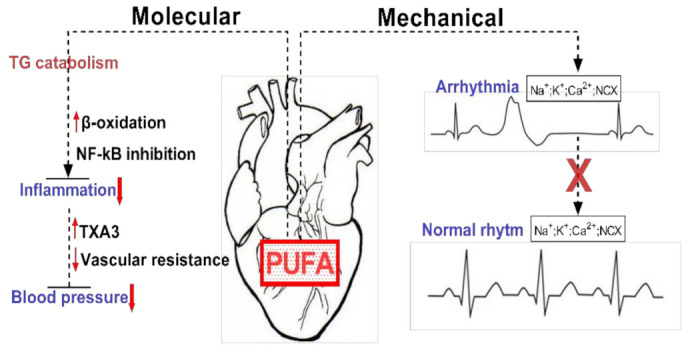
Exemplification of the molecular and mechanical implications of PUFAs in cardiac function. PUFAs are involved in reducing tissue inflammation by inducing TG catabolism via increased β-oxidation and NF-kB inhibition. Mechanically they can restore cardiac rhythm by inhibiting ion channels and exchangers.

Given the importance of FAs for ensuring the proper function of the cardiovascular system, a permanent supply of FA sources becomes critically important. The omega-3 (ω-3) fatty acids, including docosahexaenoic acid (DHA) and eicosapentaenoic acid (EPA), are predominantly present in fish and marine products, with the highest concentrations in fish oils [9]. More importantly, the mechanisms (Figure 2) by which dietary FAs (EPA, DHA) exhibit their cardioprotective properties as effective modulators for preventing cardiovascular risk factors are of considerable importance [4].

As such, a constant supply of essential FAs will contribute to the proper function of the cardiovascular system. A proper function allows the system to perform its critical role of supplying nutrients and oxygen to the cells, tissues, and organs, as well as taking away the metabolic waste by-products and removing CO_2_ from the body. The main risk factors leading to cardiovascular diseases are cardiac arrhythmia, increased blood triglyceride (TG) levels, high blood pressure, small high density lipoprotein (HDL) content, endothelial dysfunction, heart rate variability, and parallels with thrombosis and inflammation [4,10]. The ω-3-PUFAs modulate numerous molecular pathways with physiological roles [10]. Recently, it has been concluded that ω-3 FAs have an anti-inflammatory effect, through the modification of TLR4 signaling pathway activation, by suppressing dimerization in cellular membranes, leading to the inhibition of TLR4 expression and further mitigation of meta-inflammation, cardiovascular disease, and risks of diabetes myelitis 2 in obese humans [11,12]. Previous studies also highlighted the ability of PUFA to inhibit many inflammatory biomarkers associated with cardiovascular risk factors, including the generation of eicosanoids (e.g., prostaglandins (E2), thromboxane (B2, A3/prostacyclin), and leukotrienes (B4)) derived from arachidonic acid (n6-ω FA). They are also involved in the regulation of T-helper 1 lymphocyte reactivity, reduction of human adhesive molecule expression (E-selectin, P-selectin, ICAM-1, and VCAM-1), and leukocyte–endothelial adhesive interconnections, and in the decrease of pro-inflammatory cytokines (TNF, IL-1β, IL-6, and IL-8) and pro-inflammatory metalloproteinases (2 and 9) [13] [4,9,11,12,14]. The above-mentioned effects of PUFA intake mitigate the risks of coronary heart disease and cardiovascular disease events, as well as slightly alleviating the risks of coronary disease, stroke, and mortality [15]. Several molecular and physiological mechanisms have been described for PUFA, and below we describe, not only their molecular implications, but also some of the clinical effects.

### 2.1. First, Biological Mechanism Suggests that the Cellular Organelles and Membranes Are Controlled through Their Lipidic Composition 

A high dietary intake of ω-3 fatty acids results in the appropriate accumulation of these FAs in cellular membranes and circulating lipids [11]. Therefore, the engulfment of ω-3 PUFA by cellular membranes can impact the fluidity and biophysical characteristics of lipid rafts, which act together as modulators of protein functions and are also involved in signaling pathways [14]. Furthermore, EPA and DHA induce anti-inflammatory effects through the disturbance of nicotinamide adenine dinucleotide phosphate (NADPH) oxidase activity, which in turn blocks the TLR4 recruitment for lipidic rafts [11]. Specifically, cell membranes enriched with ω-3 PUFA disrupt the recruitment and initiation of toll-like receptor-4, which triggers anti-inflammatory effects via down-regulation of the NF-κB pathway. Similarly, the incorporation of ω-3 PUFA by cellular lipid membranes modulates ion channels (e.g., sodium (Na^+^) and L-type calcium (Ca^2+^)), including Na^+^ – Ca^2+^ exchangers. Previously, various in vivo and in vitro studies showed that ω-3 PUFA could influence the electrophysiology of atrial and ventricular cardiomyocytes by alleviating their excitability, and cytosolic fluctuations of calcium, especially in damaged or ischemic cells that are prone to depolarization and arrhythmia [10]. Recently, in one study, the heart's electrical activity or Long QT Syndrome was described as mediated by the involvement of PUFAs [16]. PUFA analogues modulated the capacity of different voltage-gated ion channels (NaV, CaV, and KV). However, the molecular mechanism was not identified by the authors.

### 2.2. Second, ω-3 PUFA Interact Directly with Proteins and Membrane Channels 

The direct modulation of sarcolemma ion channels or GPR120 receptor, also known as free fatty acid receptor 4 (FFA-4), was previously linked with antiarrhythmic and anti-inflammatory capacity [2]. Furthermore, ω-3 FAs modify the membrane fluidity potential and consequently change ion transportation. A molecular anti-inflammatory mechanism occurs when EPA or DHA activate the expression of GPR120 and subsequently release β-arrestin-2 into the plasma membrane, which is then bonded to GPR120 [11]. Additionally, the binding complex of β-arrestin-2 and GPR120 is furtherly recruited into the cell cytoplasm and is therefore coupled with TAK1-binding protein (TAB-1), which deteriorates the connection between TAB-1 and kinase activated by the growth factor β (TAK-1). Furthermore, this association leads to a decreased TAK-1 activation and attenuated activity of the IKK-β/NF-κB and JNK/AP-1 signaling pathways, respectively. The coupled complex of TAB-1and TAK-1 is a merging point for stimuli induced by the toll-like receptor 4 (TLR4) signaling pathway, including tumor necrosis factor receptor (TNFr) [17,18]. Similarly, a possible cardioprotective mechanism may be attributed to the activation of protein kinases B such as AKT, previously recognized as interplaying with biochemical pathways known as reperfusion injury salvage kinase (RISK) [19]. When the onset of reperfusion is initiated, these kinases bestow cardio-protective effects via mitochondrial permeability transition pore (mPTP) opening suppression. Moreover, in a murine model, DHA was reported to inhibit the opening of mPTP, inducing a drop of infarct size, through an unidentified mechanism [20].

### 2.3. Third Mechanism Reffers to Regulation of Protein Expression with Pro-Inflammatory Potential

Omega-3 PUFA can directly regulate the expression of proteins with pro-inflammatory potential in various tissues, through different types of nuclear receptors, such as the peroxisome proliferator-activated receptors (PPAR-α, β, δ, γ) that mitigate NF-κB activation, retinoid X receptors (RXRs), and liver X receptors (LXR-α and LXR-β), including transcription factors such as hepatic nuclear factor 4 (HNF-4α and HNF-4γ). Furthermore, ω-3 PUFA interactions with nuclear receptors are modulated via cytoplasmic fatty acid-binding proteins. The role of these FA binding proteins is to deliver the fatty acids directly inside the nucleus, to interact with specific receptors. Such receptors are important regulators of lipid metabolism and glucose/insulin homeostasis, which are vital cell functions associated with CVD [21] 

### 2.4. A Fourth Mechanism Refers to the Release of Phospholipids by Cytosolic Phospholipase A2 (cPLA2) 

PUFAs are enzymatically converted to eicosanoids via enzymes such as cyclooxygenase (COX), lipoxygenase (LOX), and cytochrome P450 (CYP450). CYP450 can synthesize EPA, and DHA-derived mono-epoxides (MEFAs) were previously reported to exhibit a potent biological activity that can mediate CVD, due to vasodilatory and anti-inflammatory, and ion channel modulatory properties [10,22]. In addition, CYP-derived epoxyeicosanoids sustain the cellular viability of cardiomyocytes, inhibit pro-inflammatory genes under different stress environments, and enhance mitochondria function [23]. Dietary intervention with EPA/DHA can induce anti-arrhythmic and cardioprotective effects via the mediation of the CYP-epoxygenase/sEH enzymatic pathway [23]. Interestingly, an earlier study affirmed that PUFA-derived MEFAs were approximately 1000-fold more effective in decreasing calcium overloading in rat myocytes than their parental ω-3 LC-PUFAs (EPA and DHA). The soluble epoxide hydrolase (sEH) is able to metabolize epoxygenated fatty acids (EpFA) from the degradation of PUFA [24]. Elevated contents of EpFA in the body induce many valuable effects for medicating different diseases, including cardiovascular diseases. The authors suggested that the fundamental mechanism of EpFA was to sustain a healthy microvasculature and preclude endoplasmic reticulum and mitochondrial stress formation [24]. For example, endoplasmic-reticulum (ER) stress is a protective mechanism that cells to surmount cellular stress and prevent detrimental mutations. The biochemical ER-stress pathway regulates cellular responses via a homeostatic mechanism that protects the cells from pathological, oxidative, or physiological stress factors. Furthermore, EpFA alleviates blood pressure, without causing hypotension as a side effect and showed promising anti-inflammatory potential by decreasing the nuclear translocation of NF-kappaβ, therefore inhibiting the transcriptional factor of inflammatory cytokines and eicosanoids [24,25,26]. Next, ω-3 FAs replace ω-6 FAs (linoleic and arachidonic acid (AA)) from the blood phospholipids located within the membrane, decrease the synthesis of AA-derived eicosanoids (prostaglandin E2), and, conversely, increase prostaglandins derived from ω-3 PUFA [10,11]. For instance, leukotrienes and prostaglandins are high reactive biological molecules, considered to behave as pro-inflammatory agents, and are involved in different pathologies, including atherosclerosis and other inflammatory conditions [1]. Furthermore, it is important to emphasize that many ω-6 FAs are inclined to trigger inflammation, while the ω-3 FAs have been demonstrated to alleviate inflammation.

### 2.5. A Fifth Mechanism—Involvement in the Production of Anti-Inflammatory Molecules

Metabolites derived from enzymatic actions of COX and lipoxygenase (LOX) promote the generation of novel specialized pro-resolving mediators (SPMs), such as resolvins or protectins, with additional anti-inflammatory effects to ω-3 PUFA [27]. Likewise, as previously observed, LOX-5 metabolites, and a resolvin E1 (RvE1) derived from the EPA, switch on the ChemR23 receptor and suppress the (BLT1) receptor involved in the pro-inflammatory LTB4 signaling pathway of multiple diseases, including atherosclerosis [27,28]. Several previous studies reported that the ω-3 PUFA enzymatically derived compounds, SPMs, are the main players in inflammation resolution programs that decrease chronic inflammation. Both EPA and DHA can synthesize anti-inflammatory and inflammation resolving mediators (e.g., protectins, maresins, resolvins, and AA-derived lipoxins) to prevent inflammation and promote healing [9,11]. In addition, these metabolites, the so-called pro-resolving lipid mediators, contribute to the resolution of inflammatory processes and aid the recovery of homeostasis after infection or injuries induced in tissues [13]. 

Moreover, dietary PUFAs can generate natural products through oxygenation processes, such as oxylipins, which play a role in immunity, vascular functions, and inflammation [29]. According to previous reports, the oxylipin profile of blood can be modified through dietary supplementation with a ω-3 fatty acid-enriched milkshake or fish oil intervention. The elevated pattern of oxylipins can be detected after approximately 2–6 h [30]. However, the main biological mechanisms of oxylipins are still under investigation. At present, more than 100 oxylipins have been identified, and the foremost known oxylipins are a group of eicosanoids derived from AA and octadecanoids generated from LA [29]. For example, epoxyeicosatrienoic acids (EETs), metabolites derived from AA, illustrated cardioprotective effects in reperfusion injury/ischemia due to vasodilatory and anti-inflammatory properties and were recently associated with alleviating cardiovascular risk in human epidemiological studies [7,29]. However, in contrast to EETs, mid-chain hydroxyeicosatetraenoic acids (HETEs) generated via allylic oxidation of AA by LOX can raise essential hypertension, indicating that they may be associated with the pathophysiology of hypertension [29]. Of the HETEs, 20-hydroxyeicosatetraenoic acid (20-HETE) inflicts vasoconstriction of swine coronary arteries, whilst the metabolites of 16-, 18-, and 19-HETEs can trigger vasodilation [8].

### 2.6. Mitigation of Adverse Effects Related to Cardiovascular Diseases

Another possible mechanism for how dietary ω-3 PUFA might mitigate the adverse effects of cardiovascular diseases arose with their potentiality to decrease the abundance of gut bacteria producing trimethylamine (TMA), a precursor of trimethylamine-N-oxide (TMAO), and which was previously reported in atherosclerotic plaque formation [14,19]. 

The suppression of atherosclerotic plaques by DHA and EPA also occurs via the decline of macrophages in atherosclerotic plaque, reductions of the atherosclerotic plaque's total volume ratio, and the reduced production of platelet-derived growth factor (PDGF) responsible for chemo-attractive effects and mitogen for macrophages and smooth muscle cells [2]. Studies have suggested that TMAO can be regarded as a predictor of mortality associated with heart failure, because of the nexus that promotes the initiation of pro-inflammatory cytokines (TNFα, IL-6, and IL-1β), activates NOD-, LRR-, NF-κB, pyrin domain-containing protein 3 (NLRP-3) inflammasome, as well as facilitates apoptosis [19]. Within the gut, a 14-day ω-3 PUFAs supplementation (600 mg/day) [31] raised the number of butyrate-producing bacteria with anti-inflammatory capacity [32], thus supporting strengthened intestinal barrier integrity, which subsequently prevents the transasation of intestinal content into the circulation [19]. It is indeed clear that the gut microbiota suffers negative changes in patients with diabetes or in obese patients, leading to reduced TMAO [33,34] levels, and making a clear connection between these changes and major cardiac events, such as myocardial infarction [35,36]. To relate these effects more closely to our study, the TMAO levels are also influenced by the diet and by the secondary effects on the microbiome [37]; however, little effect was observed on the TMAO levels [38]. On this note, it has only recently been shown that higher contents of *Prevotella* in hyperglycemic coronary are associated with high thrombus burden and TMAO in hyperglycemic patients [39].

## 3. Biological Markers, Clinical Effects, and Recommendations of PUFA

Current dietary guidelines indicate a requirement of 300–600 mg daily uptake of DHA and EPA for the primary prevention of cardiovascular events, while doses of 900–1200 mg are recommended for secondary prevention. Moreover, concentrations of 3000–4000 mg were indicated as necessary to alleviate higher levels of TG [14,40]. In Table 1 we have included the PUFAs (mostly EPA and DHA) that have been recently successfully involved in symptomatology improvement in the case of CVDs.

The previously defined “Omega-3 index” (O3I) biomarker indicates the ω-3 bioavailability and the frequency of high–low risk of death from ischemic heart diseases with the optimal suggested O3I levels to be above 8% [53]. The ω-3 fatty acids maintain and improve lipoprotein metabolism, which lowers the plasmatic TG levels by approximately 25–30% [14]. In addition, a concentration of 4 g/d (EPA + DHA or EPA-only) was concluded to be a safe and effective therapeutic option for reducing TG index and suggested for the amelioration of atherosclerotic cardiovascular disease risk [40,43]. Effective drops in blood pressure are induced with particularly high doses of ω-3 FAs [40]. Furthermore, previous findings from several meta-analyses confirmed that fish oil supplementation or fish consumption was associated with remarkable decreases in coronary heart disease mortality occurrence and mitigated fatal myocardial infarction, including sudden cardiac death in humans with cardiovascular diseases and without registered cardiovascular diseases [10]. In addition, the cardioprotective effect of EPA and DHA, and a dietary increase in alpha-linoleic acid (ALA), was also reported in ischemic heart disease mortality mitigation, including arrhythmia, and slightly alleviated the onset of CVD events [54]. 

During the initiation of atherogenesis, decreased serum profile of ω-3 PUFA contents in DPA, DHA, and EPA, and reduced u-6 PUFA concentrations in AA, LA, eicosadienoic, and γ-linolenic acid (GLA C18:3 ω-6), can be regarded as biomarkers for the oxidative-antioxidative modifications associated with coronary atherosclerosis. Such declines in ω-3 PUFA and ω-6 PUFA contents are prone to stimulate the formation of free radicals [55]. As well as ω-3 PUFA, DPA, which is a transitional product between EPA and DHA, is also involved in the resolution of inflammation, being an important precursor of lipid mediators such as isoprostanes, maresins, specific proteins, resolvins, and others [6]. However, the concentrations of DPA in foods are very low, and hence it has received comparably less attention than other ω-3 PUFAs. In addition, in the literature, DPA has not been studied as in-depth compared to other ω-3 PUFAs. Nevertheless, prior studies reported that DPA might play a valuable role in cardioprotection and was correlated to the regulation of biomarkers of metabolic diseases,, such as blood lipids, insulin sensitivity and platelet aggregation [6]. 

For example, long-chain ω-3 polyunsaturated fatty acids (VLCM ω-3 PUFAs) were reported in several studies, including human trials, for their potent TG-reducing ability, which may be regarded as a crucial pathway for the prevention of CVD [56]. The biological mechanism of TG reduction is ascribed to lessening hepatic synthesis and secretion [56]. Increased doses of VLCM ω-3 PUFA are suggested for patients with extremely elevated levels of TG for pancreatitis prevention [43,56]. Several studies demonstrated the ability to reducing diastolic and systolic blood pressure of these fatty acids [56]. Moreover, a recent trial concluded that statin therapy combined with 4 g/day of ω-3 EPA derivative (icosapent ethyl) for patients having serum TG levels of approximately 500 mg/dL showed significant reductions in ischemic event risk and cardiovascular death [44]. The icosapent ethyl, a purified and stable EPA ester, which combats cardiovascular-associated disorders, was also identified in multiple clinical studies [44,45,46,47,57]. For example, in a clinical (Reduction of Cardiovascular Events with EPA-Intervention Trial-REDUCE-IT) trial, randomized statin-supplemented patients with atherosclerosis and diabetes Mellitus history from the United States received icosapent ethyl (4 g/d), and this was shown to influence the primary composite endpoint, exhibiting a 31% relative risk reduction and 6.5% absolute risk reduction of initial ischemic events [45]. A similar decreasing tendency was observed in the secondary composite endpoint of non-fatal myocardial infarction and stroke, including cardiovascular death with a relative reduction of 31% and absolute risk reduction of 4.6% after icosapent ethyl treatment. In addition, Mason et al., in 2020, specified that besides the efficacy of EPA to lower TG content, EPA could stabilize cell membranes and was connected with statistically significant reductions, reducing by 20% deaths from cardiovascular reasons, followed by 31% reductions in sudden cardiac death and 48% decreases of cardiac arrest [13]. Additional clinical trials concluded that the supplementation of a 4 g daily dosage of icosapent ethyl to patients with previously established cardiovascular disease and experiencing elevated TG levels (despite the use of statin) significantly lowered the risk of major events, such as cardiovascular death, and decreased by 25% first, and by 31% total, ischemic events and strongly lowered the mortality rate by 30%, compared to the placebo group [44,46,47]. Recently, other studies have confirmed the protective outcomes of icosapent ethyl [43,48]. Based on a promising efficacy, the Food and Drug Administration (FDA) recently approved icosapent ethyl as a therapeutical agent to reduce CVD-associated risks [56].

It can be concluded that the elevated serum levels of palmitic, stearic, oleic, and linoleic acids are associated with the formation of atherosclerotic plaques in coronary arteries [58]. Moreover, alteration of serum fatty acid profile lifts the oxidative-inflammatory vascular biomarker concentration and lipoprotein-associated phospholipase A2 (Lp-PLA2) [58].

## 4. Dietary Sources of PUFA

The dietary sources of ω-3 PUFAs were previously reviewed [10]; however, in Table 2 we provide an updated review of PUFA types and sources.

### 4.1. Marine Sources

Omega-3 fatty acids are generally found in oily fish, lean meat, green leafy vegetables, tunicates, and different seeds [59,60,69,70]. Marine dwellers are a rich source of biologically active components, such as lipids, with a potent anti-inflammatory capacity, involved in alleviating cardiovascular risks. It has previously been shown that EPA and DHA statistically reduce TG concentrations and regulate the total cholesterol by greatly increasing high-density lipoprotein cholesterol (HDL-C) levels and decreasing low-density lipoprotein cholesterol (LDL-C) [71]. Several studies have also reported that ω-3 PUFAs improve endothelial function, directly influencing arterial stiffness by reducing the pulse wave velocity and ameliorated arterial compliance [14,41,72,73]. All these positive effects, including reducing hypercholesterolemia, were observed when fish oil was used [74]. Consumption of marine-derived ω-3 PUFAs is known to reduce TG levels leading to lower risk of cardiovascular morbidity and mortality [42]. This effect was shown in clinical trials, where EPA treatment of patients with high levels of TG (200 and 500 mg/dL) led to a decrease in high-sensitivity C-reactive protein (hsCRP), and oxidized LDL-C (oxLDL), lipoprotein-associated phospholipase A2 (Lp-PLA2), and lessened the proportion of AA-to-EPA conversion, in respect to control groups [42]. Moreover, in vitro work, using human endothelial cells, indicated that EPA-enriched HDL suppressed cytokine-stimulated expression of VCAM-1 and enhanced the production of resolvin E3 (RvE3). The authors concluded that EPA enriched HDL particles could exert cardioprotective effects by generating anti-inflammatory lipid metabolites and improving cholesterol efflux [75]. Other studies also showed that EPA supplementation induced downregulation of genes related to CVD, such as hypoxia-inducible factor 1 (HIF-1), and cAMP-responsive element protein 1 (CREB-1) gene expression, including the generation of inflammation-involved mediators, such as IL-1β and TNF-α [42]. Both EPA and DHA can suppress the expression of inflammation-associated genes via the nuclear peroxisome proliferator-activated receptor (PPARα/γ) [7]. 

Another source is represented by *Halocynthia aurantium*, an edible ascidian from South Korea, that is well-known for having a higher content of fatty acids such as eicosatrienoic acid ω-3 (ETA), EPA, and DHA [59]. It has been observed that is responsible for increasing NO and prostaglandin E2 (PGE2) production, without causing any cytotoxicity, and also for enhanced anti-inflammatory and immune responses [59]. Furthermore, a fraction of fatty acids was shown to regulate immune-related genes, causing a remarkable downregulation of iNOS, IL-1β, IL-6, COX-2, and TNF-α, as well as inhibiting the secretion of inflammatory biomarkers (COX-2 and PGE2) in LPS-stimulated RAW264.7 cells. This effect is important, as only IL-6 is known for being involved in initiating the generation of C-reactive protein (CRP), a biological marker for determining the risk of upcoming cardiovascular diseases and systemic associated inflammatory processes [74]. 

Similar effects were detected in the case of Australian marine seafood know for its high content of PUFAs, which notably suppress NO and TNFα production in RAW 264.7 macrophages [61]. Specifically, the cephalopod molluscs *Sepioteuthis australis* and *Octopus tetricus* displayed the highest ω-3 contents and prominent anti-inflammatory activity in vitro. Moreover, the authors explained that the extracted bioactive lipid fraction and the anti-inflammatory activity were higher than in the control reference product Lyprinol^®^, a nutraceutical with a potent anti-inflammatory activity obtained from green-lipped mussel (*Perna canaliculus*). According to previous studies, green-lipped mussel extracts have been found to reduce iNOS expression and NO production in a LPS-induced RAW 264.7 cell line, regulate NF-κB and MAPK, and suppress TNFα in LPS-induced human THP-1 monocytes [61,62]. 

Another interesting source is krill (*Euphausia superba*), a small marine crustacean from the Antarctic Ocean. It has received increased attention due to its nutraceutical potential and due to its high levels of lipids and phospholipids (39.29–80.69%), and for having a high content of (ω-3) PUFA, mainly represented by EPA and DHA [64,76]. It is important to point out that fish oil, including krill oil supplements, are different carriers of EPA and DHA. So far, fish oil, as compared with krill oil, was recently mentioned by researchers to show less efficacy in normalizing hyperlipidaemia [76]. The higher bioavailability of krill oil can be correlated with other valuable antioxidant compounds, including astaxanthin, tocopherols, vitamin A, and flavonoids, and was reported to enhance the absorption efficacy in the gut, as well as in the brain [77]. Several previous in vivo and in vitro studies showed that krill oil exhibits anti-inflammatory, anti-obesity, and anti-diabetic effects, and could function to alleviate cardiovascular diseases and other illnesses [64,76]. However, the exact molecular mechanisms are still not well-elucidated.

### 4.2. Plant Sources

Following ingestion, the alpha-linoleic acid (ALA) of vegetable sources (e.g., hemp, flax, walnut, and algae) is slowly metabolized in long-chain polyunsaturated ω-3 fatty acids (EPA and DHA) [78]. However, aquatic organisms, notably fatty fish, accumulate high amounts of plankton and algae-derived EPA and DHA, with promising effects that may serve as a first source of such fatty acids. Patients under increased cardiovascular risk that received regular diets over one year with a higher proportion of nuts, legumes, virgin olive oil, and fish tended to show enhancements of HDL functionality [70]. For example, the walnut profile of ω-3 fatty acid and ω-6 to ω-3 displayed a 4:1 ratio and was suggested to have the most suitable health-inducing proportionality compared to other vegetables/plant oils [79]. In Table 3 we present some of the most common plant sources of PUFA, with their respective proportions of ω-6 and ω-3.

The bioavailability of plant associated PUFA in the gastrointestinal tract is an important variable for PUFA to be effective in reducing CVDs and their related risks [81]. In this context, it has been shown that incorporating fish, flaxseed, and algal oil, as an emulsion, in a yoghurt matrix was proven to be an efficient alternative for n3 FA delivery and increased bioavailability. Several cardioprotective characteristics were assigned to flaxseed [82]. Flaxseed and its oil were reported to possess antihypertensive effects, with the main biological mechanism ascribed to the oxylipin fraction that inhibits soluble epoxide hydrolase (seH) activity through its ω-3 ALA content [82]. The activity of seH is responsible for the generation of cytotoxic and inflammatory oxylipins such as dihyroxyoctadecenoic acids. 

Similarly, the antiatherogenic mechanism of flaxseed was interconnected with its ability to repress the expression of proliferation cell nuclear antigen (PCNA), IL-6, and VCAM-1, which indicates its anti-inflammatory and antiproliferative efficacy [82]. The ALA content in flaxseed could modify the fatty acid structure of platelet lipids; hence, raising EPA and DHA levels in the membrane by demonstrating an antiplatelet capacity. These elevations were stated to facilitate the production of suppressive pro-aggregatory species of thromboxane A2 and anti-aggregatory PGI3. Furthermore, flaxseed can restore vascular function by improving neovascularization, consequently promoting heart functions through a compound called secoisolariciresinol diglucoside (SDG) [82]. This compound was shown to significantly reduce the occurrence of arrhythmias in an in vivo model of mice myocardial infarct when rodents were fed with 10% milled flaxseed and 4.4% supplemented flax oil enriched with ALA or flax SDG (0.44%). Moreover, after histological investigations, the dietary treatment resulted in a significantly reduced infarct rate, caused a smaller dilation of the left ventricle, and alleviated myocardial fibrosis and TNF-α levels compared to the control groups [66].

The latest progress made in deciphering the novel molecular mechanisms involved in heart function has revealed evidence of the influence of endogenously encoded microRNAs (miRNAs) on cardiovascular disease and health. It was concluded that several microRNAs (e.g., miR-1, miR-29b, miR-133a, miR-133b, and miR-135a) play a valuable role in cardiovascular pathological processes, such as fibrosis and apoptosis arrhythmias after myocardial infarction. For example, in the context of myocardial infarction, the pronounced expression of miR-1 was linked with arrhythmias, upregulation of miR-133 was associated with regulation of apoptosis in cardiomyocytes, and the inflammatory responses of the myocardium were shown to decrease the frequencies of arrhythmias and promoted cardiac reprogramming. Meanwhile miR-29b and miR-135a was interlinked with the regulatory activity of cardiac fibrosis [83]. Upregulation of miR-133 expression with interventions with dietary flaxseed was recently suggested to play an important role in promoting cardiac restoration. Upregulation of microRNAs (miR-24, miR-27, miR-126, and miR-133) has also been observed in patients with atherosclerotic plaque instability, as well as rupture in patients with asymptomatic carotid artery stenosis (ACAS). This could mainly be evidenced in patients with pre-diabetes, indicating that the miRs could represent new biomarkers of plaque instability, especially in patients with pre-diabetes versus normoglycemics [84]. In patients with diabetes, cardiovascular diseases represent one of the major causes of death, as they are highly exposed to plaque instability and have higher SGLT2 (sodium-glucose cotransporter 2) expression, inflammation, and oxidative stress. It has been reported that lower level SGLT2i-treated patients with type 2 diabetes presented lower inflammation and ion and oxidative stress, thus indicating a more stable plaque phenotype [85]. As hyperglycemia can have pro-atherogenic effects, we can conclude that miRs can indeed be responsible for plaque instability and rupture in ACAS patients with pre-diabetes, which emphasizes the importance of controlling their action in ACAS patients. Given the direct link between the pericoronary fat expression of SGLT2 and over-inflammation, it has been shown that reducing the pericoronary fat levels through metformin therapy led to the amelioration of clinical outcomes and improved the clinical outcomes of pre-diabetes patients [86]. Metformin therapy is characterized by reduced levels of inflammation, oxidative stress miR-195, and miR-27 [86]. Previous studies also demonstrated that ω-3 PUFAs defended cardiomyocytes after myocardial infarction induction and caused downregulation of three pro-apoptotic miRNAs and upregulation of nine anti-apoptotic miRNAs [87]. In light of this, flaxseed exhibited a cardioprotective action in a recent in vivo study [83]. The study concluded that dietary interventions with flaxseed oil modulated the expression of microRNAs associated with significant cardioprotective outcomes by mitigating artificial myocardial infarction. The administration of dietary flaxseed protected against small spontaneous cardiac infarcts by normalizing the ischemic lesion frequency, followed by cholesterol-lowering ability and enhancement of systolic and diastolic function in a JCR:LA-cp rat model [88].

More recently, stearidonic acid (SDA), commonly known as “pro-eicosapentaenoic acid”, from botanical sources has emerged as a sustainable ω-3 source and was reported to efficiently accumulate tissue levels of EPA and DPA in contrast to ALA, due to the specific ability of SDA to bypass the D6-desaturase (D6D) rate-limiting step [89]. The highest abundance of SDA content was recently reported for oils extracted from the seeds of Echium (*Echium plantagineum*), Hemp (*Cannabis sativa*), Blackcurrant (*Ribes nigrum*), and *Buglossoides arvensis*. In a recent in vitro human endothelial cell model, pre-treatment with 50 μM of plant SDA reduced the expression and production of ICAM-1 in EA.hy926 cells [65]. Moreover, 50 μM concentrations of plant SDA and ALA, including marine DHA, decreased the adhesion of THP-1 monocytes on EA.hy926 cells. A more pronounced anti-inflammatory activity was observed in EPA and DHA, either notably depressing the production of monocyte chemoattractant protein-1 (MCP-1), IL-6, IL-8, or meaningfully decreasing COX-2 protein expression. SDA fatty acid appears to be less studied in the literature [89]. Thus, the results of many studies are not sufficient to conclude about significant cardio-protective outcomes.

### 4.3. Uncommon Sources

Among dairy products and dairy fats, yak butter was reported as a valuable dietary source of CLA, four times higher than other kinds of butter, and has been associated with anti-carcinogenic, antihypertensive, and anti-atherosclerotic actions, as well as with inhibiting the development of osteoporosis [68]. Compared to other essential FAs, CLA can act as a strong antioxidant and is able to effectively protect structural lipids against free radicals and different reactive oxygen species. 

## 5. Biological Mechanisms of MUFA in Cardioprotection

The monounsaturated fatty acids (MUFA) are mainly reported in the literature as abundantly present in extra virgin olive oils (EVOO) and are associated with a beneficial role in cardiovascular protection [74,90]. The consumption of one tablespoon per day of EVOO was associated with reductions in cardiometabolic mortality and contrasts with butter or margarine, which are linked with increased mortality [91]. This effect is probably caused by the high content of MUFA, especially oleic acid (18:1 n-9), oleuropein, and hydroxytyrosol [74,92]. Compared to other types of FAs, oleic acid is less susceptible to oxidative processes and is better known for its role in reducing heart tissue inflammation [74,93]. The anti-inflammatory effect was linked to the MUFA content, to the high HDL-C concentrations and reduced free circulating cholesterol and TG levels, as well as diminishing HDL-c oxidation, thereby improving the glucose homeostasis and the fluidity of cellular membranes [74,94]. Moreover, diets rich in MUFAs and enriched with polyphenols reduce the production of pro-inflammatory cytokines, regulating oxidative stress and subsequently alleviating chronic inflammation [74,95]. The antioxidant effect was attributed to reducing Nox-2 activation and the release of soluble E-selectin/VCAM-1 [96]. 

MUFAs can also express epigenetic effects, but this depends on the dosage and the specific subtype of FA. In human THP-1 cultured monocytes, one of the MUFA representatives, namely oleic acid (OA), was found to be responsible for the induction of global hypomethylation and for the ability to ameliorate inflammation patterns [97]. In more recent studies, oleic acid caused the upregulation of let-7b specific miRNA expression and, consequently, had anti-inflammatory properties [98]. The epigenetic landmarks altered by SFA (palmitic acid) and MUFA (palmitoleic acid) were related to the metabolic pathways involved in the dysregulation of lipid metabolism, glucose misbalance, and obesity [6,99]. However, DNA methylation and histone deacetylation arbitrated to PUFAs was associated with cardioprotective effects and normal weight. Moreover, oleic acid was reported to prevent palmitic-SFA-advanced mitochondrial dysfunction, refine the blood lipid profile, and enrich LDL particles with oleic acid, which will decrease the presence of LDL particles in the walls of the arteries; hence, decreasing atherosclerosis risk [6].

The replacement of SFAs with MUFA and PUFA has been significantly associated with a decrease of metabolic syndromes, which is another cluster of concurrent CVD risk factors [100]. Moreover, an increased intake of MUFA, or the substitution of SFAs by MUFA, was recently reported to enhance insulin signaling and fat distribution, and regulate post-prandial cellular oxidative stress in patients suffering from metabolic syndrome [98]. One of the reported biological mechanisms engaged in oleic acid (oleate) efficiency may lay behind the ability to preclude NF-κB or JNK-1/2m activation in feedback to TNF-α or palmitate [98]. For example, in endothelial cells, oleic acid decreased ICAM-1, monocyte chemotactic protein 1 (MCP-1), hindered the proliferation triggered by palmitate, angiotensin II, and apoptosis caused by TNF-α, and inversely raised the levels of e-NOS expression resulting from pro-inflammatory cytokines [98].

Apart from olive oil, similar effects on anti-thrombotic activity, control of hypertension, endothelial functioning, platelet aggregation, and blood coagulation were observed in coconut oil [101]. Virgin coconut oil showed a beneficial role in lipid metabolism, and the effect on cardiovascular protection was ascribed to its capability to promote macrophage-specific reverse cholesterol transport (RCT). RCT measurement was suggested as a useful biological marker of cardiovascular risk depiction and played a valuable role in anti-atherogenesis [102,103]. Likewise, in a recent in vivo comparative study, virgin coconut oil, due to a vast content in lauric acid, appeared to mediate RCT via up-regulation of the hepatic mRNA expression of SRB1 and ABCA1, certainly through the PPAR-α - LXR-α - ABCA1 signaling pathway in macrophages [103].

## 6. Relevant Clinical Effects Following Diets Rich in MUFA

The post-prandial phase was described as being in strong conjunction with oxidative stress-related inflammation, as an outcome triggered by LPS that can induce deleterious effects on cardiovascular health [104]. In a recent study, patients with impaired fasting glucose that received a lunch supplemented with 10 g of EVOO had a visible mitigation of the post-prandial LPS phenomenon [104]. On the contrary, the patients who did not received EVOO displayed an elevated expression of serum oxidative biomarkers such as sNox2-dp, LPS, Apo-B48, and ox-LDL. Furthermore, this study concluded that EVOO mitigated oxidative stress-associated inflammation was induced by LPS via downregulation of Nox2-derived peptide (sNox2-dp) interlinked with TLR4.

In the PREDIMED clinical trial, it was concluded that individuals with high cardiovascular risks showed decreased LDL atherogenicity indices associated with oxidation after receiving a traditional Mediterranean diet supplemented with 1 l/week of virgin olive oil compared to a low-fat control diet. [105]. A separate trial indicated that EVOO supplementation to obese patients reduced body fat, diastolic blood pressure, and IL-1β compared with the control group [106]. Additionally, a ten year large cohort ATTICA prospective study has preliminarily reported a meaningful inverse association that supports olive oil consumption for preventing primary CVD risks and suggested that plasma fibrinogen played a mediating key role in this correlation [107].

## 7. Dietary Sources of MUFA

Oils extracted from canola, apricot, avocado, safflower, mustard, almond, peanut, and olives were recently reported as having low SFA but rich MUFA contents and were suggested as suitable for inclusion in diets due to their cardioprotective properties [108]. Moderate amounts of MUFA were detected in soybean, sunflower, rice bran, cottonseed, grapeseed, corn, and sesame oils. Nuts such as walnuts, pistachio, and macadamia are rich sources of heart-protective PUFAs and MUFAs, of which ω-9 oleic acid (18:1 ω-9) was reported as the predominant mono-unsaturated ω-9 FA, followed by ω-6 LA (18:2 ω-6) and ω-3 ALA [79]. The oleic acid (18:1 n-9) fraction from different mushroom species can range from 1.0 to 60.3% [67]. The LC-MUFA from marine oils decreased the formation of atherosclerotic lesions and cholesterol efflux, and the altered gene expression of parameters in relationship with lipid metabolism, energy, and inflammation in several tissues [109]. In Table 4 we summarize some of the types and sources of MUFAs.

## 8. The Implication of Free Fatty Acids, Saturated Fatty Acids, Long-Chain Saturated Fatty Acids, and Short-Chain Fatty Acids in Cardiovascular Diseases

Increased blood levels of free fatty acids (FFAs) usually appear in patients with cardiovascular diseases, obesity, and type-two diabetes (T2DM), and their occurrence is linked with depressed endothelium or insulin-dependent vasodilation, weakened nitric oxide (NO) production, and insulin signaling, with amplified inflammation and oxidative stress [110]. A diet rich in ω-3 or ω-6 FAs administered in the right dosage and formulation was suggested to exhibit protective effects on the endothelium and can be regarded as an alternative method for the mitigation of endothelial dysfunction [110]. Further evidence suggests that dysfunction of the vascular endothelium and inner lining of the blood vessels is an important factor in the pathogenesis of atherosclerosis. The most common long-chain fatty acids (LCFAs) that are present within the Western diet are myristic acid (14:0), palmitic acid (16:0), and stearic acid (18:0), and are suggested to possess a higher risk for coronary heart disease, compared to SCFAs or MCFAs [110].

Recent studies have shown that palmitic acid mediates apoptosis of the vascular smooth muscle cells (VSMC), advances cellular and inflammatory responses in cardiac fibroblasts, thereby activating TLR4 and NLRP3 inflammasome, raising mitochondrial ROS levels and decreasing the functionality of cardiac fibroblasts [110,111]. Palmitate in microvascular endothelial cells (line EOMA) appeared to stimulate NLRP3 inflammasome activation, disrupting endothelial tight junction (TJ) proteins such as zonula occludens-1 (ZO-1) and zonula occludens-2 (ZO-2) [112]. The authors explained that the increase of free fatty acids promotes high mobility group box-1 (HMGB-1), responsible for TJ disruptions, and increases the endothelial paracellular permeability associated with endothelial injury.

Circulating fatty acid-binding protein (FABP-4), secreted from various cells (e.g., vascular endothelial, adipocytes, and macrophages), delivers fatty acids to the organs and acts as a bioactive molecule for different target cells, such as smooth muscle cells, endothelial cells, and macrophages [113]. Elevated circulatory FABP-4 levels were correlated with hypertension, obesity, atherosclerosis, left ventricular diastolic dysfunction, heart failure, and DMT2 [113,114]. In addition, the fundamental FABP-4 was independently linked with modifications in carotid intima-media thickness (CIMT), which is a surrogate marker for atherosclerosis prediction, and in earlier studies, serum FABP-4 levels were shown to predict long-term cardiovascular events and mortality [115,116]. Previously, in vitro adipocyte treatment with ω-3 FAs ethyl esters (EPA and DHA) appeared to partially diminish FABP-4 expression [114].

The dietary intake of lipids can induce modifications in the expression of TLRs [11]. A meta-analysis concluded that increased concentrations, in a dose-response manner, of trans fatty acids were correlated with a higher risk of CVDs [117]. For example, patients exposed to meals of increased caloric value, including high carbohydrate and lipid content, will have induced changes in TLR during the post-prandial period, caused by elevated TLR2 and TLR4 expression in blood mononuclear cells [11]. Thus, such changes in TRL in the post-prandial period may be responsible for the development of diabetes mellitus type 2 (DMT2), obesity, and cardiovascular disease physiopathology. Stimulation of the inflammatory response via the TLR4 signaling pathway was observed in the case of lauric acid (C12:0) and palmitic acid, which are representatives of the SFA family. Previous in vitro findings indicated that in macrophages, lauric, palmitic, and stearic acid enables COX-2 expression via a NFκB mechanism that leads to TLR4 activation, whereas such effects were not detected for MUFA and PUFA [11]. Similarly, MUFA and PUFA notably reversed the pro-inflammatory outcomes induced by lauric acid. Furthermore, SFA can also induce an inflammatory response by enabling the TLR1, TLR2, and TLR6 signaling pathways, as previously described elsewhere [11].

Some of the microbial synthesized compounds, such as those produced by Gram-negative bacteria and known as lipopolysaccharides (LPS), can trigger an inflammatory response via intercellular surface interactions with specific receptors from the immune cells, such as neutrophils and macrophages [11]. Subsequently, a study concluded that the TLR4 signaling pathway enhances the exposure to LPS and SFAs; both relevant features for obesity development. More interestingly, ω-3 FAs were reported to act against TLR and its agonists, such as LPS.

The long-chain fatty acids (LCFAs) are absorbed less efficiently within the gastrointestinal tract than medium-chain fatty acids (MCFAs) [118]. LCFAs are packed in ultra-low-density lipoproteins (chylomicrons) and circulate via the lymphatic system, permitting an elevated uptake by adipose tissue. This aspect is extremely important, since it has been shown, for example, that at the level of breast glands the adipose tissue is associated with major adverse cardiac events (MACEs) in pre-menopausal women [119]. In addition, LCFAs require a particular carnitine shuttle, essential for transport into mitochondria [118]. On the other hand, MCFAs are transported through the portal vein to the liver and promote rapid oxidation. In contrast, MCFAs do not require a carnitine shuttle to be transported into the mitochondria and are engaged in fatty acid oxidation. A recent study suggested that when MCFA are substituted for dietary long-chain TG, the metabolic routes might increase energy expenditure and satiety, thus providing weight control [118].

Arterial hypertension and resulting impacts on the organ sequelae are prone to arise from T cell-mediated inflammatory diseases. A previous in vivo study emphasized that SCFAs play an important role in cardiovascular protection [120]. For example, in rodents, propionate administration decreased cardiac hypertrophy, fibrosis, atherosclerotic lesions, and sensitivity to cardiac arrhythmias [120]. The authors specified that such protective effects are caused by propionate, which could influence T helper cell homeostasis. Their study concluded that oral supplementation with propionate, including their precursors, might play a positive role as a nonpharmacological preventive strategy for hypertensive subjects, to prevent damage to target organs [120].

Explicitly, SFAs and very-long-chain saturated fatty acids (VLSFAs) are considered joint biomarkers of dietary and metabolic processes [121]. The main sources of VLSFAs arrive from foods such as macadamia nuts and peanuts, including canola oil, or they can even be synthesized endogenously form shorter-chain SFAs, as in the case of behenic acid C22:0 and lignoceric acid C24:0 [121,122]. VLSFAs are mainly composed of sphingomyelins and ceramides. Specifically, ceramides are involved in apoptosis, which is essential for the pathophysiology process in heart failure. Long-chain saturated FAs (palmitic acid 16:0) in combination with ceramides can promote apoptosis, whilst VLSFA (lignoceric acid 24:0) with ceramides functions to counteract and protect against apoptosis [121]. The explanation for this lies in the deleterious effects of ceramide with palmitic acid that formed channels in the mitochondrial outer membrane, which triggers membrane permeabilization and apoptosis. However, ceramide in combination with lignoceric acid grasps the inner and outer membranes; therefore, disrupting the palmitic acid-mediated channels by averting induced permeabilization and apoptosis. The exact interrelation between VLSFA and heart failure has not yet been elucidated and determined. However, increased circulating VLSFAs were recently reported and associated with decreased risk of DMT2, atrial fibrillation, and coronary disease [121,123].

## 9. Impact of Bioavailability

The bioavailability of ingested nutrients is very inconsistent, as it is dependent on various factors, such as the food matrix; the relationship between different food ingredients, which can affect absorption properties; the physicochemical composition (e.g., chemical binding form); and the metabolization rate [124]. Until recently, the bioavailability of EFA has been considered of minor importance. However, given the above mentioned health improvement outcomes, the bioavailability of EFA has become of major importance, especially in relation to the absorptive capacity [125]. On the negative side, the acquisition of beneficial properties from omega 3 fatty acids is restricted due to poor water solubility and weak oral bioavailability. For example, fish oils are prone to lipid oxidative processes due to their high level of unsaturation, which can affect their regular consumption by promoting a fishy and rancid odor, and even promote adverse effects for health [126,127,128]. Notably, in fish oils, DHA and EPA are naturally bound by default in triacylglycerides, while a wide variety of fish oil capsules are composed of fatty acids bound in ethyl-esters and re-esterified triacylglycerides. In contrast, the best bioavailability was indicated in the case of phospholipid-bound omega-3 fatty acids from krill oil formulations, characterized by incorporation of DHA and EPA into the plasma, with re-esterified triacylglyceride fish oils and followed by ethyl esters [129].

Over recent decades, research has focused on improving the bioavailability of ω-3 fatty acids, in order to increase the bioavailability of EFAs using targeted delivery systems. Advanced encapsulation technologies can overcome certain difficulties, involving the controlled release of hydrogels, microgels, emulsion-based droplets, nanostructured lipid carriers, and liposomes [130,131]. Adopting an appropriate encapsulation methodology can drive significant improvements in the water dispersibility, chemical stability, and bioavailability of ω-3 fatty acids. Using nanoemulsified and microemulsified formulations provides smaller oil droplets, which can significantly enhance the physicochemical stability and bioavailability, as previously observed in several biologically active oils, including fish oils, essential oils, sunflower, and olive oils in combination with hydrophilic nanocarriers [131,132,133]. Moreover, small droplets of ω-3 PUFA are easily delivered to the body via cellular membranes, boosting blood plasma and erythrocyte concentrations [134].

One approach for achieving a better protection against ω-3 oxidation is tuna oil incorporation in a WPI-GA (whey protein isolate gum arabic) complex, followed by drying processes utilizing spraying and freeze-drying to obtain solid microcapsules [126]. A recent study achieved oxidation stability by encapsulating chia seed oil in a matrix obtained from ionic gelation using a *Salvia hispanica* mucilage combined with calcium and alginate [135]. A second option is the incorporation of ω-3-rich phospholipids derived from marine oils in the hydrophobic nonpolar tails of phospholipids to synthesize liposomes that could confer an enhanced oxidative stability [131]. To our knowledge, marine-based liposome delivery systems were previously proven to remarkably enhance the bioavailability of ω-3 fatty acids in respect to standard fish oils [136].

Previous studies suggested that lipid-based formulations can improve the bioavailability of ω-3, as well as their ingestion with high-fat meals, which can improve their bioavailability [128]. It was also recommended that the ingestion of emulsified forms of omega-3 oils should have higher bioavailability patterns because such supplements can be enriched with absorption enhancers that advance the permeability for the epithelium cells [131]. Moreover, emulsions provide the greater surface area required by pancreatic lipases for adsorption. In the case of two Epanova formulations, ω-3 carboxylic acids and ω-3 free fatty acids, a higher bioavailability was documented during fasting conditions and low fat supply, where the activation of pancreatic lipases was skipped and an additional digestive step was not necessarily for intestinal enterocyte absorption [126,137].

Alternatively, to avoid the requirement of additional high-fat meals, absorption accelerating technology recently developed a novel safe and self-micro-emulsifying delivery system (SMEDS) with the formula (PRF-021), which can accelerate and ease emulsification fabrication and droplet formation for the targeted delivery of the aqueous milieu to the gastrointestinal tract [138]. Meanwhile, in the intestinal lumen, the droplets became more susceptible to the digestive processes, where they can effectively release and diffuse the embedded concentrations of EPA and DHA for better absorption into the mucosal cells [138]. In human trials, it was shown that over time, EPA and DHA ethyl esters were prominently better absorbed in plasma in the PRF-021 formulation compared with the control baseline treatment composed of EPA and DHA ethyl ester concentrate [138]. Overall, the authors concluded that data from two clinical trials illustrated that ethyl ester of EPA and DHA assembled with SMEDS formulation showed a greater absorption and six-times greater efficiencies in contrast to the reference supplements following pharmacokinetic evaluation.

Recently, a nanoemulsion was designed, aiming to improve DHA bioavailability from ALA-rich linseed oils [139]. The improvement of bioavailability of DHA was based on the inoculation of linseed oil in combination with curcumin embedded in a phospholipid core material (Lipoid™), which resulted in a significantly elevated level of DHA in the serum and tissue lipids of animals. In a separate study, a new formulation based on in vivo investigations demonstrated a significant increase in the bioavailability of DHA in the plasma. In this research, ω-3 tocopheryl phosphate mixture (TPM) was prepared from DHA and EPA (DHA 500 and EPA 50 mg/g) combined with tocopheryl phosphate dissolved in food-grade canola oil (OA 61%, LA 21%, and ALA 11%), which contributed to doubling DHA levels in plasma after 4 h, compared with the control treatment with omega-3 oil (DHA 500TG) without the addition of tocopheryl phosphate mixture [128]. The before-mentioned availability of alpha-tocopheryl phosphate (TPM) as a new lipid excipient for oral delivery for poorly aqueous soluble agents was also correlated with a superior bioavailability in earlier studies, due to compelling safety characteristics, demonstrated to provide cardioprotective effects and mitigate atherosclerosis and inflammation [128,140,141]. Conclusively, the encapsulation and formation of microcapsules will improve the bioavailability of FAs by creating bioactive compounds which can be easily incorporated in food matrixes, to ensure a protected delivery via the digestive system.

## 10. Conclusions

This review discussed several recently identified biological mechanisms and specifically focused on how dietary ω-3 fatty acids can have positive cardiovascular effects, depending on their bioavailability. The authors have used data available from in vitro and in vivo studies, including clinical studies, to describe and characterize the signaling pathways involved, in parallel with the connection between FAs and their lipid mediators and receptors. This approach was taken to pave the way for new avenues for therapeutic approaches that will benefit the resolution of inflammation in cardiovascular diseases. Finally, recent clinical trials provided new evidence regarding the benefit of EPA administration in patients treated with statins and previously diagnosed with cardiovascular diseases, by reducing the occurrence of cardiovascular risk factors and mortality.

## Figures and Tables

**Table 1:** Essential PUFAs associated with the relief and improvement of CVD clinical symptomatology.

Fatty Acid	Dose	Type of Trial	Mechanism	Outcomes	Refs
60% EPA and 40% DHA	1800 mg/day	Hypertensive patients with hypertriglyceridemia	Ability to incorporate into phospholipid membranes by partially replacing arachidonic acid as an initial substrate to produce anti-inflammatory eicosanoids	Improved arterial stiffness and endothelial function	[41]
EPA-only	1.8–4 g	Patients with greater increased TG levels	Mechanisms that inhibit free radical propagation	Decreased hsCRP, oxLDL, Lp-PLA2 and AA-to-EPA conversion	[42]
EPA + DHA or EPA-only	>3 g/d	Patients with hypertriglyceridemia	Suppression of SREBP-1 activity, inhibition of its activation via posttranslational mechanisms, and degradation of its active form. Increased peripheral triglyceride clearance and a reduction in intrahepatic fatty acid pools	Reducedtriglyceride index and was suggested for amelioration of atherosclerotic cardiovascular disease risk	[43]
EPA	4 g/d	Patients with established cardiovascular disease or with diabetes and other risk factors	Not identified, but it is possibly due to the stabilization or regression of coronary plaque and differences in hsCRP levels	Significant reductions in ischemic events risk and cardiovascular death	[44]
		Patients with atherosclerosis and diabetes Mellitus history		31% relative risk reduction and 6.5% absolute risk reduction of initial ischemic events	[45]
		Patients with previously established cardiovascular disease and experiencing elevated triglyceride levels		Significantly lowered the risk of major events such as cardiovascular death and decreased by 25% for first, subsequent and by 31% total ischemic events and strongly lowered the mortality rate by 30%	[44,46,47]
		Patients with coronary atherosclerosis	Possibly through the improvements in lipid oxidation, inflammation, plaque volume, membrane stabilization, and dyslipidaemia	Significant regression in low-attenuation plaque volume	[48]
EPA and pitavastatin	1800 and 4 mg/day	Patients with coronary heart disease	Probably through anti-inflammatory function	Significantly reduced coronary plaque volume and reinforced plaque stabilization	[49]
	1800 and 2 mg/day	Patients with acute coronary syndrome	Not identified, but possibly through anti-arrhythmic, anti-inflammatory effects	Reduced adverse cardiovascular events following percutaneous coronary intervention	[50]
EPA and DHA	1.86 and 1.5 g/day	Subjects with stable coronary artery disease on statins	Not specified	Abrogated the progression of fibrous coronary plaques	[51]
46% EPA and 38% DHA	4 g/d	Patients with familial hypercholesterolaemia	Probably due to the influence of arterial elasticity, by enhancing nitric oxide production, AA displacement in membrane phospholipids, increased production of n-3 derived eicosanoids	Improved large arterial elasticity and arterial blood pressure independent of statin therapy	[52]

**Table 2:** Dietary sources of PUFA.

Relvevant PUFA Sources		
Marine Sources		
Tunicate (*Halocynthia aurantium*)	ETA, EPA and DHA	[59]
Chub mackerel (*Scomber japonicus*)	DHA, EPA and SDA	[60]
Squid (*Sepioteuthis australis*)	DHA, EPA	[61]
*Octopus tetricus*	DHA, EPA	
Australian sardine (*Sardinops sagax*)	DHA, EPA, DPA	
Atlantic salmon (*Salmo Salar*)	LA, DHA, DPA	
Eastern king prawn (*Penaeus plebejus*)	DHA, DPA, EPA	
Green lipped mussel oil complex (*Perna canaliculu*)	EPA, DHA	[62]
Brown seaweed (*Fucus vesiculosus*)	EPA	[63]
Krill (*Euphausia superba*)	EPA and DHA	[64]
**Plant Sources**		
Hemp (*Cannabis sativa)*	ALA, LA	[65]
Flaxseed (*Linum usitatissimum*)		[66]
Blackcurrant (*Ribes nigrum*)	SDA	[65]
Corn gromwell (*Buglossoides arvensis*)		
Mushrooms *spp*.	LA, ALA	[67]
**Other Sources**		
Yak butter	CLA	[68]

**Table 3:** Most common plant sources and their PUFA content (ω6 and ω3) [80].

Source	Total (%)	*ω*6 (%)	*ω*3 (%)
Black cumin	15.99	58.74–61.10	0.21–0.99
Chia	23.66	16.67–23.43	44.50–68.84
Flax	28.73	12.78–74.09	2.40–59.70
Hemp	38.10	58.76–62.48	14.55–18.76
Perilla	29.98	10.08–18.35	53.14–65.60
Pumpkin	20.98	32.00–55.35	Traces 0.58
Quinoa	3.29	44.90–56.64	3.80–9.57

**Table 4:** The common types of MUFAs in different types of seeds (per 100 g) and in extracted oil [80].

Source	Seeds (%)	Oil (%)
Black cumin	5.99	23.00–24.91
Chia	2.31	5.59–10.95
Flax	7.53	11.30–24.13
Hemp	5.40	9.92–13.81
Perilla	4.93	12.58–17.00
Pumpkin	16.24	23.58–44.12
Quinoa	1.61	19.40–35.00

## Data Availability

Not applicable.
